# Transcutaneous spinal cord stimulation modulates quiet standing in healthy adults: stimulation site and cognitive style matter

**DOI:** 10.3389/fnins.2024.1467182

**Published:** 2024-09-11

**Authors:** Natalia Shamantseva, Olga Timofeeva, Varvara Semenova, Irina Andreeva, Tatiana Moshonkina

**Affiliations:** Pavlov Institute of Physiology, Russian Academy of Sciences, St. Petersburg, Russia

**Keywords:** postural control, postural strategy, spinal cord, transcutaneous electrical stimulation, neuromodulation, healthy subjects, cognitive style

## Abstract

The study explored the effects of transcutaneous electrical spinal cord stimulation (tES) on postural control. Subjects were divided into field-dependent (FD) and field-independent (FI) groups according to their cognitive style. FD subjects use an exteroceptive afferent stream for spatial orientation, while FI subjects use an interoceptive stream. In darkness, vertical posture is maintained by head-trunk stabilization in FD subjects and by independent movements of body segments in FI subjects. Previously, we showed that tES at the L1-L2 vertebral level decreased postural stability in FD subjects. Now, stimulation was applied at the T11-T12 vertebral level (midline, above the left or right dorsal roots). Quiet standing was assessed using stabilometry in 18 FD and FI participants. Participants stood on a force platform in soundproof chamber with eyes closed during tES. Midline and left tES significantly improved postural stability by up to 28% in FD participants, while posture did not change significantly in FI participants. Pronounced differences between the effects of T11-T12 and L1-L2 stimulation are associated with selective topographical activation of proximal and distal leg muscles during tES of the lumbar enlargement. This study highlights the importance of considering cognitive style in postural control research.

## 1 Introduction

Spinal cord (SC) serves as a central hub for coordinating locomotor tasks, integrating sensory information, generating motor commands, and adjusting movement patterns to ensure locomotor control and postural balance. SC stimulation can be used to activate multiple pathways involved in locomotor and postural control.

In animal studies, it was found that epidural electrical stimulation at the lumbar level significantly facilitated postural limb reflexes and in decerebrated cats, tonic electrical stimulation of the spinal cord facilitated weight-bearing hindlimb stepping and maintained postural stability (Musienko et al., 2010; Musienko et al., 2012). Epidural spinal cord stimulation in humans with complete motor loss after spinal cord injury enables voluntary control of leg flexion and extension as well as standing and independent walking (Gill et al., 2018). In patients with chronic paraplegia, epidural electrical stimulation of the lumbosacral spinal cord enabled volitional control of task-specific muscle activities, including standing (Grahn et al., 2017).

Transcutaneous electrical stimulation (tES) of the lumbar segments of the SC activates spinal locomotor centers, generates rhythmic stepping-like movements, and facilitates retraining of the locomotor network (Gerasimenko et al., 2015; Gerasimenko et al., 2017). Biphasic or monophasic rectangular pulses of 0.3 to 1.0 ms duration, a frequency of ~15–50 Hz filled with a carrier frequency of 5 to 10 kHz and an intensity in the range of 10 to >150 mA are used for tES. Low-current tES primarily activates low-threshold Ia large-diameter afferents, engaging locomotor spinal networks and motor axons. Studies on animals and volunteers reveal that this modulation of sensory pathways and interneurons, influenced by supraspinal input, coordinates complex motor pool activities (Gerasimenko et al., 2015).

TES of the SC offers a significant advantage in studies of the mechanisms of postural maintenance by allowing non-invasive activation of SC networks. This method facilitates the study of spinal cord neural networks in healthy individuals (Shamantseva et al., 2023; Carrera et al., 2022; Omofuma et al., 2024).

Maintaining postural balance and proper alignment of body segments with respect to gravity is a complex process in which sensory inputs from the visual, vestibular, and somatosensory systems play a key role (Ivanenko and Gurfinkel, 2018). A traditional interpretation of interindividual differences is that subjects vary in the degree to which they weigh sensory inputs (Peterka and Loughlin, 2004). Studies suggest that cognitive style influences postural control (Witkin and Asch, 1948; Witkin and Goodenough, 1981). The concept of the visual field dependence-independence cognitive style emerged as a result of the work of Witkin (Witkin and Asch, 1948). Witkin and Goodenough (1981) claimed that field-independent (FI) individuals rely on an internal frame of reference, while field-dependent (FD) individuals rely on an external frame of reference.

FD and FI subjects display different segment stabilization strategies (Wapner and Demick, 2014). Studies suggest that FD subjects rely on a visual frame of reference both for perception and postural control, whereas FI subjects would rather rely on gravito-inertial frames of reference specified by vestibular information and/or motor–proprioceptive loops. In the case of visual cue disturbances, FD subjects showed increased efficiency in hip stabilization in space and tended to have an “en bloc” functioning of the shoulder-hip unit and head-trunk unit, indicating a more integrated approach to postural control (Isableu et al., 2003). FI subjects rely primarily on appropriate non-visual reference frames for segmental stabilization strategies, making them less sensitive to postural challenges. The stabilization results provide evidence that FD subjects swayed more than FI and that darkness altered postural stability to a greater extent in FD subjects (Isableu et al., 2010). Later, it was shown that the auditory stimuli had different effects on the vertical posture of FD and FI subjects (Timofeeva et al., 2019, 2020).

Thus, tES of the SC may have different modulatory effect on the posture of FD and FI participants due to initially different joint coordination strategies.

The location of tES is critical for targeting specific neural structures in different motor pools when investigating specific neuromodulatory effects. A recent systematic review showed that the optimal locus for spinal stimulation to improve voluntary control of the lower extremities is T11-T12 vertebrae level (García et al., 2020). This spinal level corresponds to the L1-L2 spinal segments, a level at which propriospinal control of the central pattern generator is located (Dimitrijevic et al., 1998). Targeted electrical stimulation at the T11-T12 and L1-L2 vertebrae levels has been shown to recruit different populations of motor neurons through projecting sensory and intraspinal connections, leading to the facilitation of postural synergies (Gerasimenko et al., 2017). Epidural electrical stimulation promptly restored voluntary walking control in individuals with severe paralysis by specifically targeting proprioceptive circuits through the recruitment of selected posterior roots of lumbosacral enlargement of the SC (Wagner et al., 2018). The results of a study on subjects unable to stand due to severe spinal trauma showed that with targeted tES of the SC at the level of the T11 or L1 vertebrae (above the L1–L4 spinal segments) all 15 participants could independently maintain an upright posture with minimal assistance from the knees or pelvis, and seven of them could achieve this without any support (Sayenko et al., 2019). Targeted transcutaneous stimulation at T11 and L1 vertebrae levels also enhanced generation of motor patterns and enabled control of leg movements in healthy volunteers (Moshonkina T. et al., 2021).

In our previous study (*N* = 16), we showed that tES of the SC at the L1-L2 vertebrae level significantly decreased postural balance up to 30% in FD and had no effect in FI subjects (Shamantseva et al., 2023). Four experimental conditions were examined: three of them involved tES applied in the midline between the L1-L2 vertebrae and over the left or right dorsal roots of the SC at the same level and one control condition without tES. Midline and left root tES significantly destabilized posture, while right dorsal root stimulation was ineffective. We attributed this effect to an increase in joint stiffness in the lower limbs, particularly in the ankle joints induced by tES at L1-L2 vertebrae (Moshonkina T. R. et al., 2021), which are critical for postural control in FD subjects. All participants had a right dominant leg and this is likely to be the reason for the left–right asymmetry.

This study aimed to investigate the modulatory effect of the tES on quiet standing applied to the T11-T12 vertebrae level on FD and FI subjects. FI subjects use segmental stabilization, whereas FD subjects perform “en bloc” operation, meaning that their whole trunk sway in one direction without segmental compensation. It is known that stimulation at the T11-T12 level results in a lower activation threshold for proximal leg muscles, especially flexors (Sayenko et al., 2015). We hypothesized that tES applied at this site would activate proximal leg muscles and stiffen the shoulder-hip unit, modulating quiet standing in FD subjects, and probably not have a significant effect on postural control in FI subjects.

## 2 Materials and methods

The procedures and studies were conducted in the spring of 2024 in compliance with the principles of the Declaration of Helsinki and received approval from the Ethics Committee of the Sechenov Institute of Evolutionary Physiology and Biochemistry of the Russian Academy of Sciences (Minutes #2–03 dated 2024/02/26). All the subjects signed informed consent.

### 2.1 Participants

Subjects were recruited from students and colleagues who volunteered to participate. The exclusion criteria were age outside the range of 18–35 years, body mass index (BMI) outside the range of 18.5–24.9 kg/m2, scoliosis, hernias, vestibular disorders and a history of epilepsy. The study included 18 volunteers (11 males and 7 females, 27 ± 4 years). The participants’ BMI was 22.8 ± 2.6 kg/m2. Each participant considered themselves to be healthy on the day of the study. Two participants had left leg dominance, sixteen participants had right leg dominance, as determined by the ball-kick test (Paillard and Noé, 2020).

### 2.2 Procedure and tasks

The procedures, tasks, and methods are similar to those described in our previous research (Shamantseva et al., 2023). Changes in vertical posture were measured using Stabilan-01-2 force platform system (Rhythm Ltd., Russia) with StabMed 2.13 software (Sliva, 2005) placed in the center of a soundproof anechoic chamber to eliminate the effect of the audio stimuli on vertical posture (Timofeeva et al., 2019, 2020).

**Figure 1:**
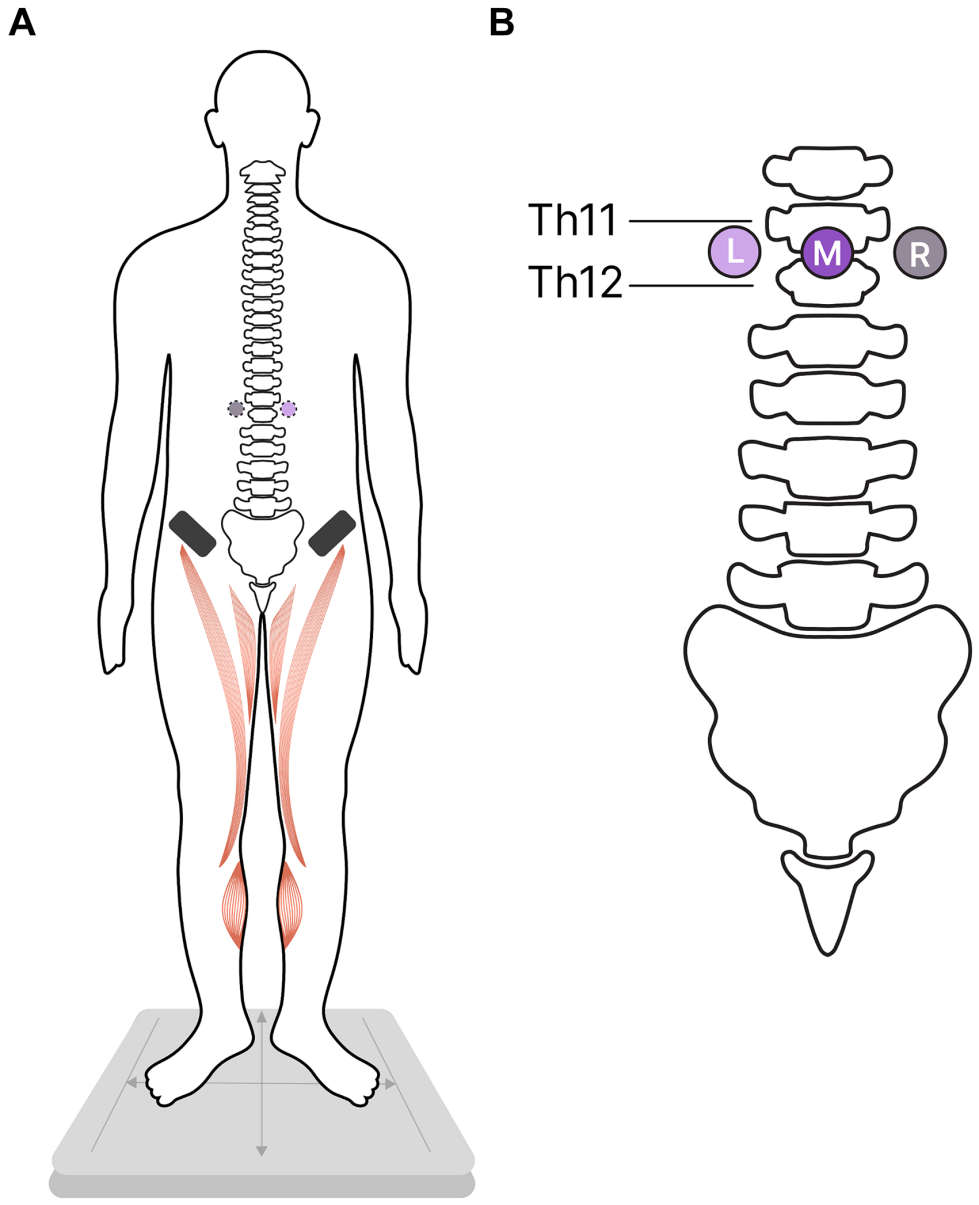
Study protocol. Participants were instructed to stand in a standard position while the transcutaneous electrical stimulation (tES) of the spinal cord was used to modulate flexor activity in one of three stimulation conditions. In the control condition, participants stood without tES. **(A)** Ventral view of the participants; activated flexors marked in red; asterisks – cathodes, black rectangles - anodes. **(B)** Dorsal view: The placement of the cathodes for tES relative to the spinal column; L, left; M, midline; R, right cathodes.

Participants stood in a standard position (heels together, toes apart, and hands down along the body) (Figure 1A) with their eyes closed under the four experimental conditions. In three of these conditions, the tES of the SC was applied at one of these loci: in the midline between the T11-T12 vertebrae and over the left or right dorsal roots of the SC at the same level (Figure 1B). Standing without tES served as the fourth control condition. For each of the four experimental conditions, the position of the participants’ centre of pressure (CoP) was recorded. Investigator was present in a chamber during recordings and controlled the subjects.

CoP recording and tES were conducted for 70 s. The analysis focused on the interval between 30 and 60 s. The initial 30 s and the final 10 s were excluded to eliminate the influence of the initial stimulation effect and the anticipation of the end of the controlled standing period.

Two independent sets, each containing four recordings, were performed. The order of the four recordings was randomized. Each set of these four conditions was considered an independent series, as a test and a retest. This approach allowed us to obtain two measurements for each participant under all experimental conditions. Short breaks were allowed between recordings. During these breaks, participants were permitted to step off or rest on the force platform.

To eliminate the potential for voluntary or involuntary effort during tES, participants were given a cognitive distraction task in all experimental conditions, which involved silently subtracting a two-digit number from a four-digit number (Woollacott and Shumway-Cook, 2002).

To determine the cognitive style of the participants the Group Embedded Figures Test modified by Gottschaldt was used (Wapner and Demick, 2014; Gottschaldt and University of California, 2012; Andreeva et al., 2018). This pencil-and-paper test is the most frequently used assessment for field-dependence/independence (Hayes and Allinson, 1994). The test participants were asked to find one of five reference figures among thirty masked figures and indicate it. The complex figures were presented one at a time. The total time of test performance was recorded. The more correctly the tasks were completed, the shorter the test completion, the more pronounced the FI.

### 2.3 Transcutaneous electrical stimulation of the spinal cord and dorsal roots

Neostym-5 (Cosyma Ltd., Moscow, Russia) was employed for tES. Stimulation was carried out at a frequency of 20 Hz using monopolar-modulated current pulses (1 ms, carrier frequency 5 kHz).

Three adhesive cathodes (2.5 cm un diameter, ValuTrode® Axelgaard Manufacturing Co., Fallbrook, CA, USA) were attached to the skin on the back: one along the midline between the T11-T12 vertebrae, and two positioned approximately 1.5 cm to the left and right of the midline electrode, along the dorsal roots of the SC between the T11-T12 vertebrae (Figure 1). Two adhesive anodes (5 * 10 cm2, ValuTrode® Axelgaard Manufacturing Co., Fallbrook, CA, USA) were placed symmetrically above the iliac crests.

Three channels were used to separately select the intensity of tES for the stimulation sites. The intensity of tES was set to the highest level that did not induce pain or discomfort. The current intensity was individually determined for each tES site.

### 2.4 Analysis

Analysed CoP parameters included: the length of the CoP trajectory, linear velocity and the root mean square deviation (RMSD) of the CoP along the frontal and sagittal axes, and the ellipse area (formulas are provided in Supplementary Table S1). Increased values of these dependent parameters indicate less stable postural control. Decreased values indicate more pronounced postural control.

Statistical analysis of the CoP parameters was performed using Analyse-it for Microsoft Excel 6.15.42024 (Microsoft Office 2021). The Shapiro–Wilk W test was used to determine the data distribution. Non-parametric statistics were used where not all data were normally distributed.

Depending on the data distribution, values are presented as mean ± standard deviation or median [1st quartile (Q1), 3rd quartile (Q3)].

The significance of differences between experimental conditions was determined using the Wilcoxon test (differences are presented as *p*-values and Z-scores) or Student’s T test (differences are presented as *p*-values and T-scores) depending on the data distribution. The Mann–Whitney U test was used to calculate the significance of the differences between the parameters of the FI and FD participants. Spearman’s correlation (*r*) was used to calculate the influence of leg dominance. The significance threshold in all tests was set at the level *p* < 0.05.

## 3 Results

### 3.1 Cognitive style: CoP parameters in control condition

Of the 18 participants, nine subjects had FD cognitive style and nine FI based on the results of the Gottschaldt test. Two groups had almost equal proportions of males and females, 5 males and 4 females in the FI group and 6 males 3 females in the FD group. The two groups did not differ in BMI (*p* = 0.21, *T* = −1.27) and age (*p* = 0.77, *T* = −0.29).

**Figure 2:**
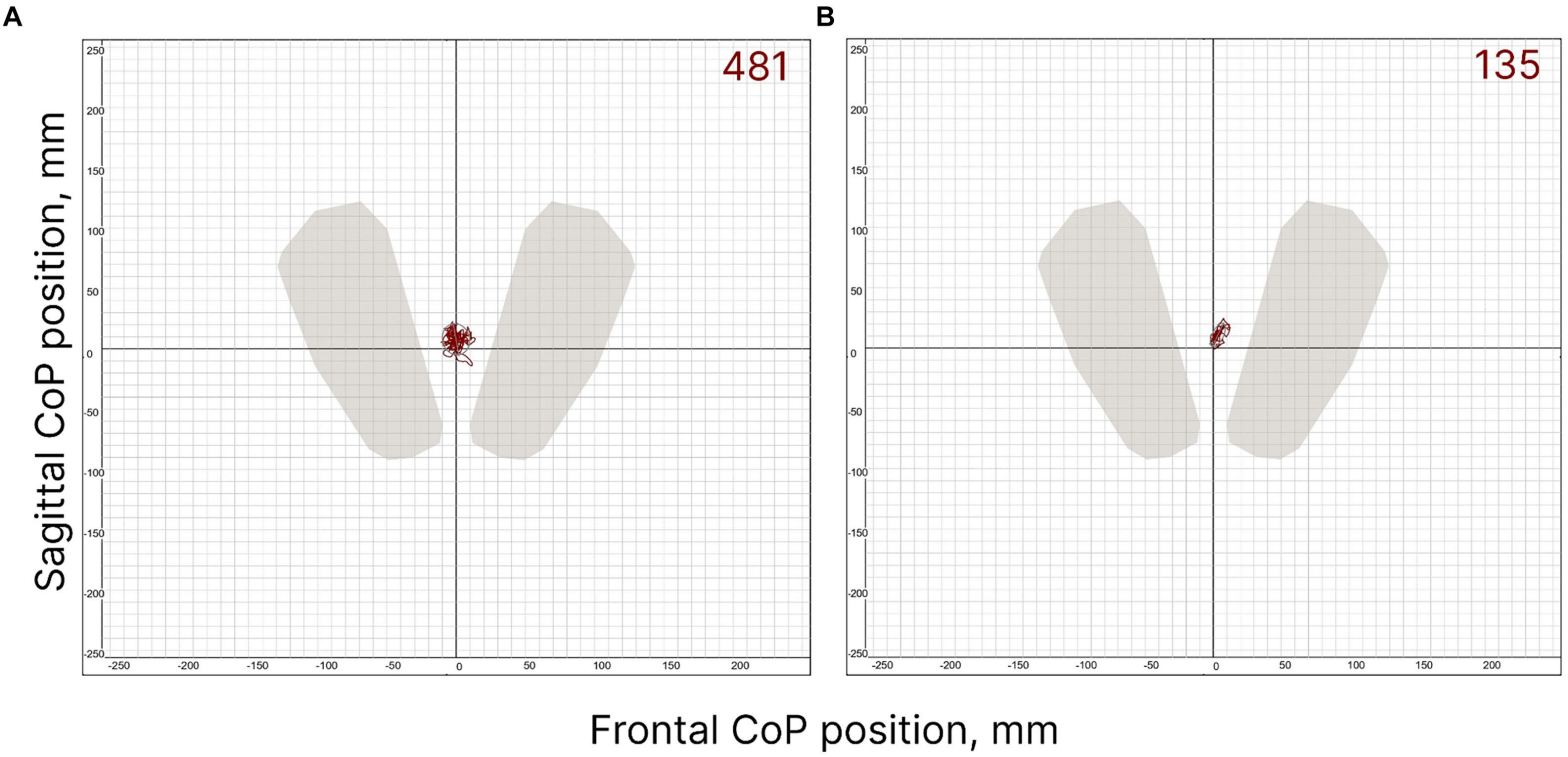
FD **(A)** and FI **(B)** subjects’ individual CoP trajectories in control condition (without spinal stimulation); the period analysed is 30 s. The value of the ellipse area is shown at the top right of the figures (mm^2^).

The postural control of the FD participants was weaker than that of the FI participants (Figure 2). Two CoP parameters were significantly different between the FI and FD groups. The length of the CoP trajectory (*p* = 0.012, *Z* = −2.5), as well as the linear velocity (*p* = 0.012, *Z* = −2.49) along the sagittal axis, were significantly higher in FD participants (Table 1). These two parameters indicate significantly greater sway along the sagittal axis in FD participants. The length of the CoP trajectory (*p* = 0.056, *T* = −1.98), as well as the linear velocity (*p* = 0.057, *T* = −1.97) along the frontal axis tended to be higher in FD participants (Table 1). Other postural parameters analyzed were not significantly different between FD and FI groups without tES (Supplementary Tables S2, 24, S5, S7).

**Table 1:** Postural parameters of the FI, FD participants significantly differed between groups.

Participants	Trajectory length_sag_, mm	Linear velocity_sag_, mm/s	Trajectory length_front_, mm	Linear velocity_front_, mm/s
FD (*n*^1^ = 18)	263 [160; 313]^*^	8.7 [7.1; 10.2]^*^	178 ± 46^#^	5.7 ± 1.2^#^
FI (*n* = 18)	161 [144; 249]	5.3 [4.6; 8.2]	109 [76; 196]	3.6 [2.4; 6.3]
All (*n* = 36)	243 [161; 294]	8.0 [5.2; 9.6]	156 ± 53	5.0 ± 1.7

^1^Number of records, tests and retests are considered for each participant. ^*^p < 0.05 relative to results of the FI participants. ^#^*p* = 0.06 relative to results of the FI participants.

### 3.2 CoP parameters with tES

#### 3.2.1 Length of the CoP trajectory

**Figure 3:**
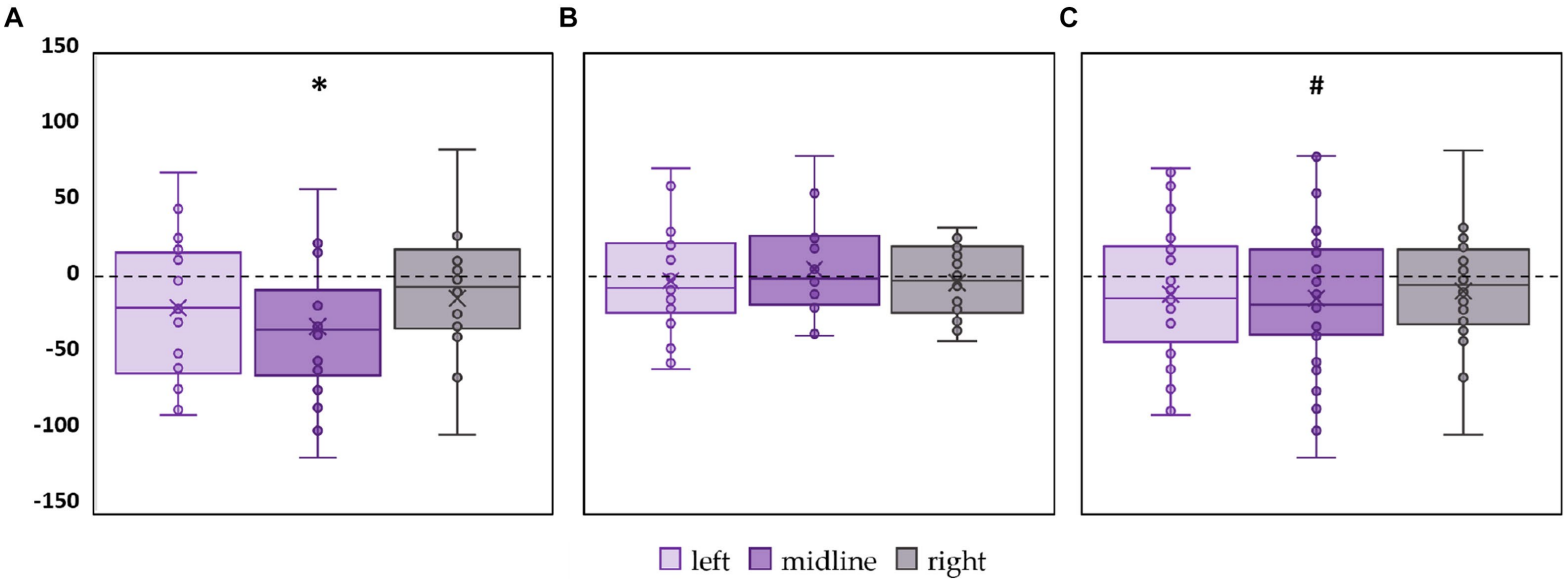
Differences in the length of the center of pressure trajectory along the frontal axis between the stimulation and control conditions, in mm. Outliers are not displayed. **(A)** FD participants; **(B)** FI participants; **(C)** all participants; *p < 0.05, #*p* = 0.09, compared to control.

CoP trajectory length along the frontal axis significantly decreased by 28% (*p* = 0.02, *Z* = −2.53) in the FD participants during the midline tES compared to the control condition (Figure 3A; Supplementary Table S2). No significant changes were obtained in FI participants between the values of this CoP parameter in the control and stimulation conditions (Figure 3B; Supplementary Table S2). In the combined group of participants this CoP parameter showed a tendency to decrease by 13% (*p* = 0.09, *Z* = −1.65) during the midline tES compared to the control condition (Figure 3C; Supplementary Table S2).

**Figure 4:**
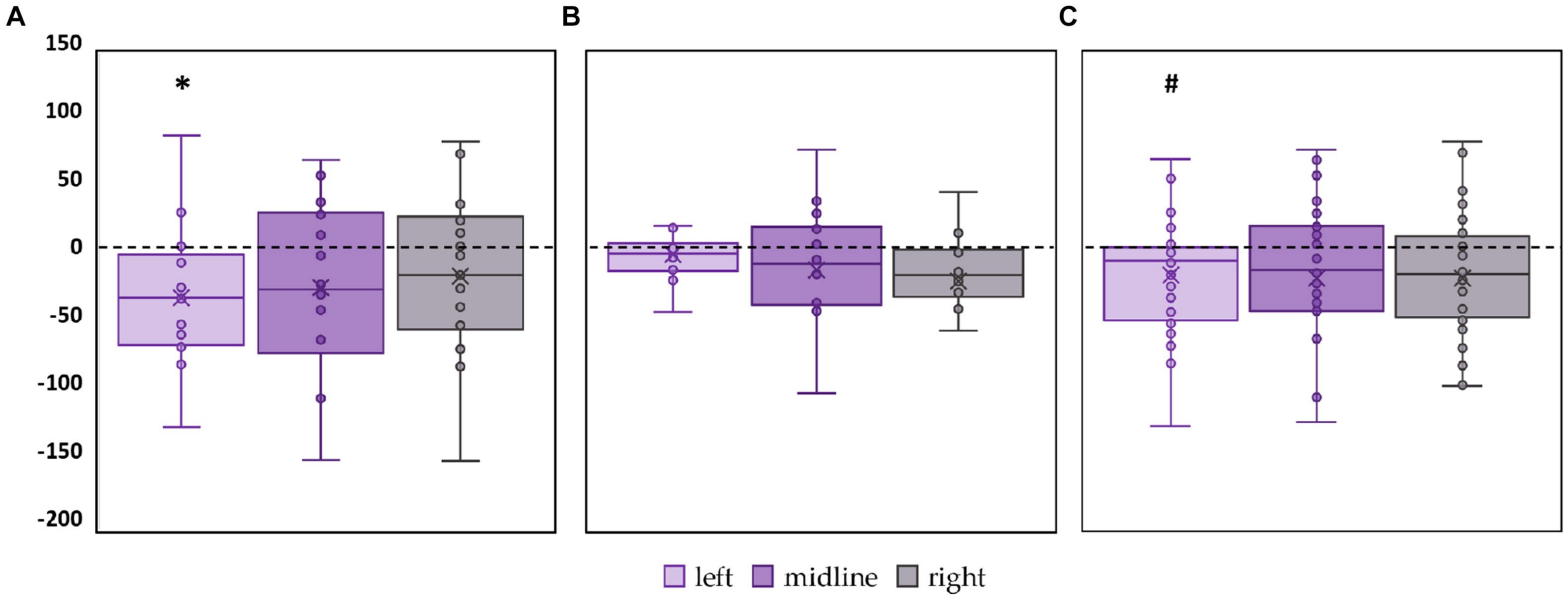
Differences in the length of the center of pressure trajectory along the sagittal axis between the stimulation and control conditions, in mm. Outliers are not displayed. **(A)** FD participants; **(B)** FI participants; **(C)** all participants; *p < 0.05; #*p* = 0.07 compared to control.

CoP trajectory length along the sagittal axis significantly decreased by 15% (*p* = 0.02, *Z* = −2.61) in the FD participants during the left roots tES compared to the control condition (Figure 4A; Supplementary Table S3). No significant changes were obtained in FI group between the values of this CoP parameter in the control and stimulation conditions (Figure 4B; Supplementary Table S3). In the combined group of participants this CoP parameter showed a tendency to decrease by 13% (*p* = 0.07, *Z* = −1.75) during the left roots tES compared to the control condition (Figure 4C; Supplementary Table S3).

#### 3.2.2 Ellipse area

**Figure 5:**
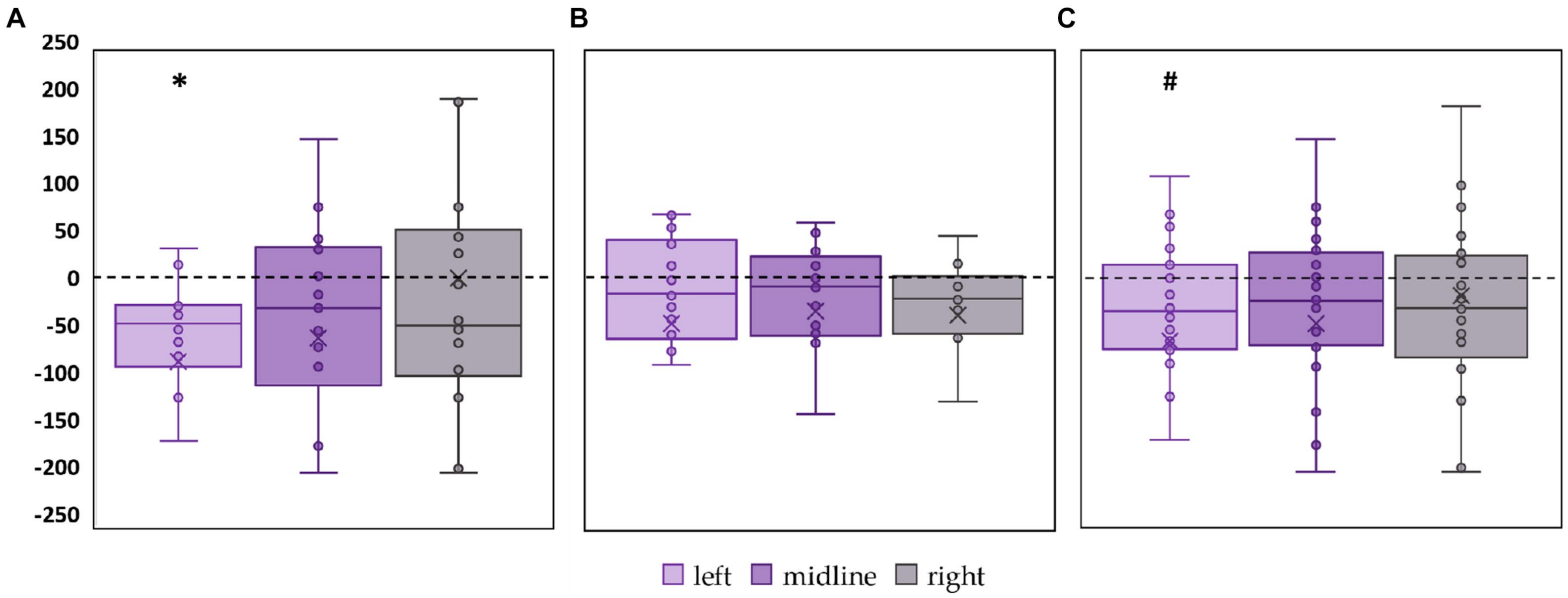
Differences in ellipse area between stimulation and control conditions, in mm^2^. Outliers are not displayed. **(A)** FD participants; **(B)** FI participants; **(C)** all participants; *p < 0.05; #*p* = 0.06 compared to control.

The left roots tES decreased the ellipse area of the FD participants by 27% (*p* = 0.01, *Z* = −2.11) (Figure 5A; Supplementary Table S4). No significant changes in ellipse area were observed in FI participants (Figure 5B; Supplementary Table S4). In the combined group, this CoP parameter tended to decrease by 23% during tES of the left root (*p* = 0.06, *Z* = −1.85) (Figure 5C; Supplementary Table S4).

#### 3.2.3 Linear velocity

**Figure 6:**
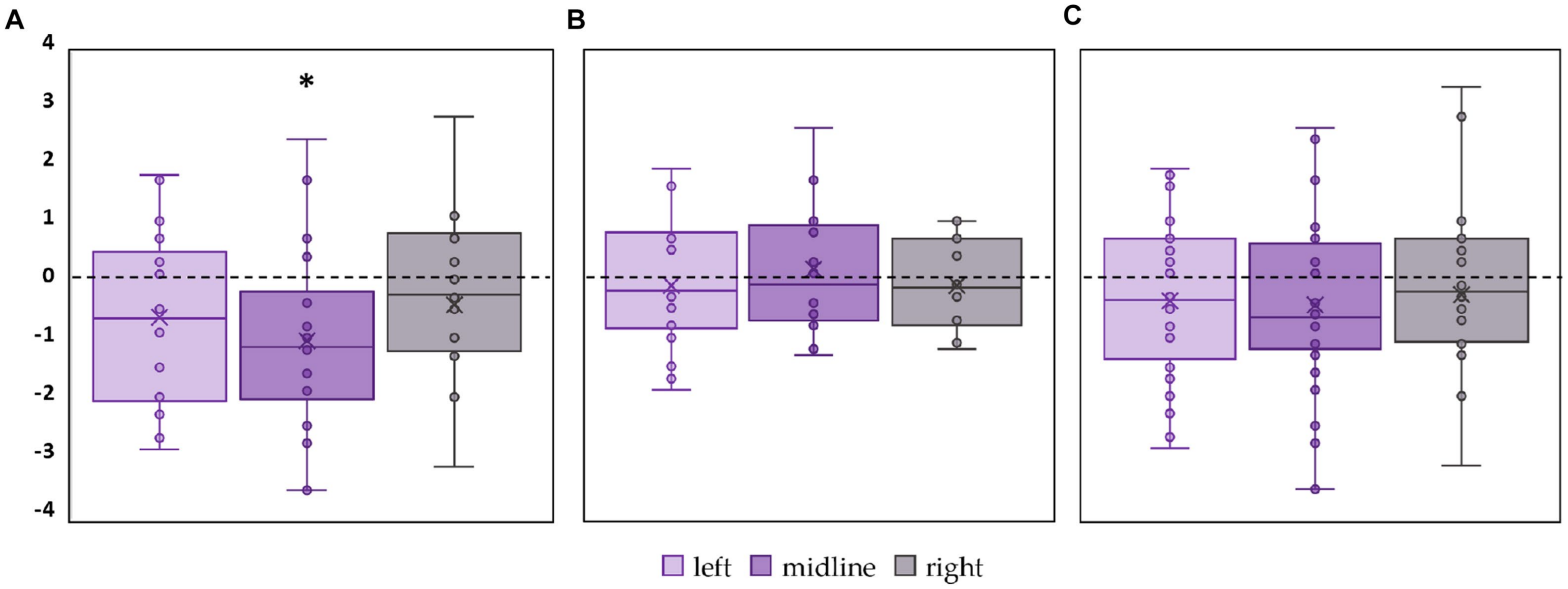
Differences in linear velocity along the frontal axis between stimulation and control conditions, in mm/s. Outliers are not displayed. **(A)** FD participants; **(B)** FI participants; **(C)** all participants; *p < 0.05 compared to control.

Linear velocity along the frontal axis significantly decreased by 28% in the FD participants during midline tES (*p* = 0.02, *Z* = −2.46) (Figure 6A, Supplementary Table S5). No significant changes in linear velocity along the frontal axis were observed in FI participants as well as in the combined group (Figures 6B,C; Supplementary Table S5).

**Figure 7:**
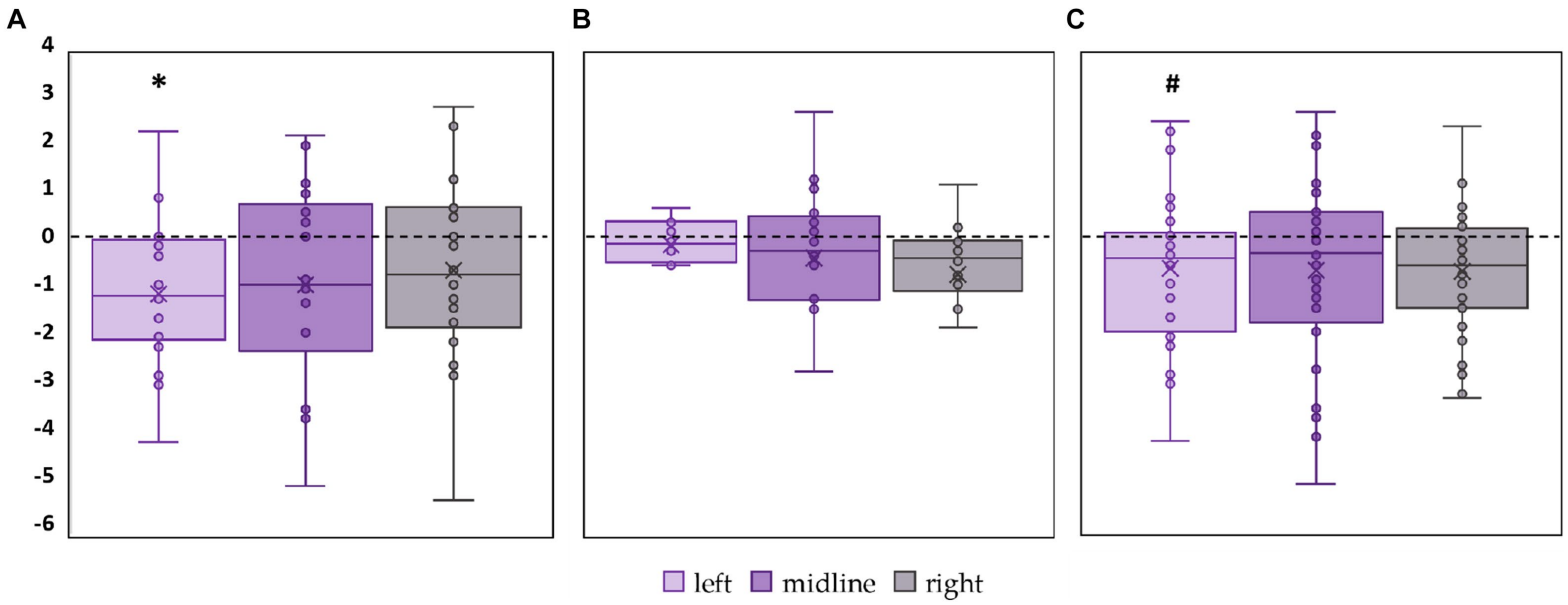
Differences in linear velocity along the sagittal axis between stimulation and control conditions, in mm/s. Outliers are not displayed. **(A)** FD participants; **(B)** FI participants; **(C)** all participants; *p < 0.05; #p = 0.07 compared to control.

Linear velocity along the sagittal axis significantly decreased by 16% in the FD participants during left roots tES (*p* = 0.02, *Z* = −2.73) (Figure 7A; Supplementary Table S6). No significant changes in linear velocity along the sagittal axis were observed in FI participants (Figure 7B; Supplementary Table S6). In the combined group, this parameter tended to decrease by 15% during left roots tES (*p* = 0.07, *Z* = −1.78) (Figure 7C; Supplementary Table S6).

#### 3.2.4 RMSD of the CoP

**Figure 8:**
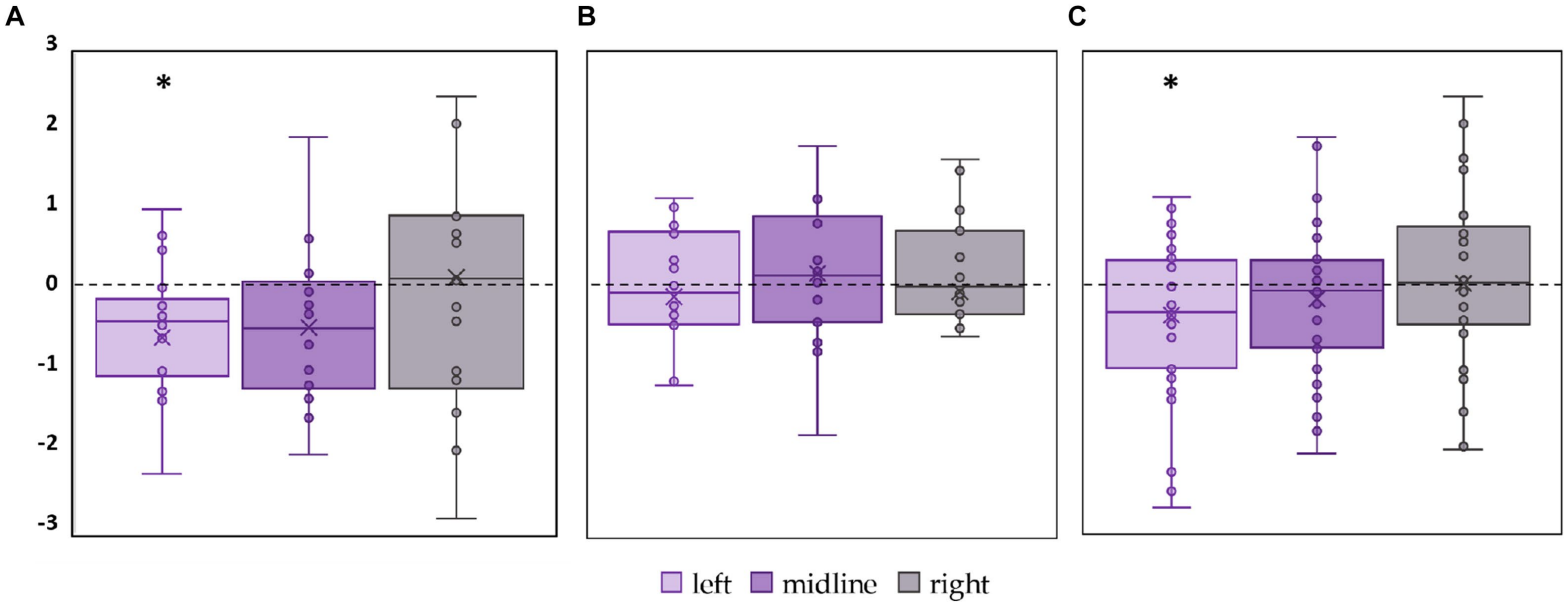
Differences between the root mean square deviation of the center of pressure along the frontal axis in the stimulation and control conditions, in mm. The outlier points that lie either below the lower whisker line or above the upper whisker line are not shown. **(A)** FD participants; **(B)** FI participants; **(C)** all participants; *p < 0.05 compared to control.

The significant decrease by 18% in RMSD along the frontal axis (p = 0.01, T = −3.29) was observed in FD participants during tES of the left roots (Figure 8A; Supplementary Table S7). No significant changes in this parameter were observed during stimulation in the FI participants (Figure 8B; Supplementary Table S7). In the combined group, RMSD along the frontal axis also significantly decreased by 12% during left root tES (*p* = 0.03, *T* = −2.19) (Figure 8C; Supplementary Table S7).

### 3.3 Current intensity

Current intensity for tES ranged from 12 to 55 mA. Intensities did not differ significantly between the FD and FI participants and the stimulation sites (Table 2). Individual variability of current intensities grouped by sex and cognitive type is shown in Supplementary Figures S1, S2. Thus, any differences in tES effects on postural parameters between the FD and FI groups and between stimulation sites are not related to current intensity. Spearman’s correlation analysis showed no significant correlation between CoP parameters in stimulation conditions and current intensities and between cognitive type and current intensities (r ≤ 0.5, *p*-value >0.05).

**Table 2:** Current intensities of the tES.

Participants	Left dorsal roots, mA	Midline, mA	Right dorsal roots, mA
FD (*N* = 9)	26 ± 9	30 ± 9	26 ± 9
FI (*N* = 9)	30 ± 13	32 ± 14	29 ± 13
All (*N* = 18)	28 ± 11	31 ± 12	27 ± 11

### 3.4 Lateral tES and leg dominance

Midline tES and left roots tES affected the posture of FD participants, who all had right dominant leg. Two participants in FI group had a left dominant leg. To evaluate the effect of leg dominance, we excluded these two subjects from the paired analysis between the control and tES conditions. No significant difference was obtained for six FI subjects with right dominant leg in any of the CoP parameters studied compared to the control values (*p*-value >0.05).

Spearman’s correlation also showed no significant correlation between CoP parameters in all the studied conditions and leg dominance (r ≤ 0.5, *p*-value >0.05).

To assess the presence of asymmetry we compared the results from left and right tES of the dorsal roots. No significant differences were observed between the left and right tES in the combined group, or separately in the FD and FI groups.

## 4 Discussion

### 4.1 Cognitive style

The results of this and our previous (Shamantseva et al., 2023) study showed that, without stimulation, participants with the FD cognitive style exhibited significantly less postural sway compared to those with the FI cognitive style. Postural recordings were made with eyes closed in the soundproof chamber, without visual and auditory anchors for standing control. The exteroceptive afferent stream provides the stability of upright standing in FD subjects, and the interoceptive afferent stream – in FI subjects. Thus, the experimental conditions were difficult for FD participants and not for FI participants.

Studies suggest that FD subjects rely on a visual frame of reference both for perception and postural control, whereas FI subjects would rather rely on gravity-inertial frames of reference specified by vestibular information and/or motor–proprioceptive loops (Isableu et al., 2003). In the absence of a total and static visual frame of reference, FD subjects have shown an increased efficiency of the hip stabilization in space strategy and an “en bloc” operation of the shoulder–hip unit. The last “en bloc” operation was extended to the whole head–trunk unit in darkness, associated with hip stabilization in space. The stabilization results provide evidence that FD subjects sway more than FI and that darkness (lack of visual information) altered postural stability to a greater extent in FD subjects (Isableu et al., 2010).

Cognitive style may determine the postural strategy humans exhibit, involving the use of muscle patterns and sensory interactions to maintain stability. The ankle strategy generates torque at the ankle joint to restore stability, while the hip strategy uses torque at the hip to counteract destabilizing forces (Nashner and McCollum, 1985; Morasso et al., 2019). Research shows that joint oscillations are attracted to either in-phase (resembling the ankle strategy) or anti-phase (resembling the hip strategy) coordination modes (Faugloire et al., 2006). Thus, FD subjects may predominantly exhibit an anti-phase coordination mode that involves hip stabilization in space. In FI subjects, each body segment contributes independently to maintaining an upright posture.

The current study revealed that tES applied at the T11-T12 vertebrae level significantly improved postural control in FD subjects but had no significant effect on FI subjects. Specifically, for the FD group, the length of the CoP trajectory and linear velocity along both the frontal and sagittal axes decreased significantly during midline and left roots tES. The ellipse area and RMSD along the frontal axis showed a significant decrease during left roots tES. In contrast, FI participants did not exhibit significant changes in any of these parameters. Similarly, we showed that tES applied at the L1-L2 vertebral level significantly altered postural control in FD subjects, but had no effect in FI subjects (Shamantseva et al., 2023). Lumbar stimulation decreased postural control in FD participants.

If we were to examine the effect of tES on the entire group of participants without defining their cognitive style, we would not have identified the stimulation effect. Significant differences would be considered random for RMSD along the frontal axis (Figure 8C). In the present study, the significance of paying attention to individual cognitive styles, which are associated with the postural strategy, is even more evident than in our previous work (Shamantseva et al., 2023), where the destabilizing effect of stimulation on the entire group of participants was almost identical to that in the FD group. Therefore, when designing postural studies and analyzing the results, it is important to consider the cognitive style of the participants. Cognitive styles reflect the type of afferent stream, exteroceptive or interoceptive, that is dominant in providing the stability of an individual’s upright posture, since the coordination of body segments in upright standing differs between FD and FI subjects.

### 4.2 Spinal segment-specific tES

In our previous work, we showed that tES of the SC at the L1-L2 vertebrae significantly decreased postural balance by up to 30% in FD subjects (Shamantseva et al., 2023). Four experimental conditions were examined: three of them involved tES applied in the midline between the L1-L2 vertebrae and over the left or right dorsal roots of the SC at the same level and one control condition without tES. All participants had right dominant leg, and only midline and left roots tES significantly destabilized the posture, also significant difference between the tES of left and right dorsal roots was obtained. We attributed postural destabilization of the FD participants during L1-L2 tES to the increased coactivation leading to the stiffness of ankle joints (Moshonkina T. R. et al., 2021). The increased stiffness of the leg joints during tES increased the overall stabilization of the joints, and the instability of the upright posture was similar to that shown when the joints were mechanically stabilized (de Freitas et al., 2009).

Studies on the postural effect of tES suggest that the stimulation can modulate the integration of voluntary descending commands and sensory signals, altering muscle activation during postural tasks, even in neurologically intact subjects (Carrera et al., 2022). A recent study showed that tES applied midline between the L2-L3 vertebrae initially increases muscle activity in specific muscles, particularly during forward perturbations, which led to a decrease in balance performance in that direction (Omofuma et al., 2024).

It has been shown that tES is capable to selectively activate sensory and motor roots depending on spinal levels (Sayenko et al., 2015; Krenn et al., 2013). Stimulation delivered along the rostrocaudal axis of the lumbosacral enlargement in the supine position resulted in a selective topographical recruitment of proximal and distal leg muscles. Responses to single pulses were higher in vastus lateralis and rectus femoris during tES applied to the T10-T11 vertebrae levels and lower during stimulation at the T12-L1 level. Stimulation at the L1-L2 level led to the selective activation of distal leg muscles such as tibialis anterior and medial gastrocnemius (Sayenko et al., 2015). Stimulation at the T11-T12 and L1-L2 vertebrae has been shown to recruit different populations of motor neurons through projecting sensory and intraspinal connections, leading to the facilitation of postural synergies (Gerasimenko et al., 2017).

The neuromodulatory effect of tES highly depends on spinal segment. Lateral stimulation at T11 engaged the neural circuits controlling flexion, whereas lateral stimulation at L1 primarily recruited the circuits controlling extension. Spatiotemporal T11-L1 stimulation enhanced generation of motor patterns and enabled control of leg movements (Moshonkina T. et al., 2021).

It was shown in healthy volunteers walking on a treadmill that unilateral tES of the posterior roots of the SC at the level of the L1 vertebrae during the stance phase causes an increase in coactivation of the shin muscles on the side of stimulation (Moshonkina T. R. et al., 2021).

In this study we applied tES in the midline between the T11-T12 vertebrae and over the left or right dorsal roots of the spinal cord at the same level in order to activate motor pools of proximal flexor muscles of the lower limbs. Stimulation at the T11-T12 vertebrae level straightens back curvature and increases trunk stability in individuals with spinal cord trauma (Rath et al., 2018; Keller et al., 2021). We attribute the stabilizing effect of tES applied to the T11-T12 vertebrae to the activation of the hip flexors, which leads to trunk stabilization, resulting in fixation of the hip-shoulder unit. This effect induced an increase in postural stability in the FD participants. In the FI subjects, the body segments moved independently from each other during upright standing, and for this reason tES at T11-T12 did not affect postural balance.

In summary, we assume that in the absence of visual frame of reference, followed by cognitive task that may inhibit supraspinal postural regulation (Pitts and Bhatt, 2023), FD subjects respond to spinal modulation by performing “en bloc” operation, stiffening the shoulder-hip unit. FI subjects may perform segmental balancing that compensates spinal modulation.

### 4.3 Asymmetry and leg dominance

We also investigated two factors that could affect postural parameters: the leg dominance and lateral position of the stimulation electrode.

In our previous study with tES applied to L1-L2 all 16 participants were right leg dominant and in the FD participants, most of the CoP parameters obtained during tES of the left roots were significantly greater than those obtained during the stimulation of the right roots (Shamantseva et al., 2023). In this study, two out of eight FI participants had left dominant leg and this did not affect the CoP parameters nor did we obtain significant differences between left and right roots tES.

Dominant limbs often exhibit more refined and efficient motor control (Sherwood, 2014). This is due to greater neural activity and possibly denser neural connections on the dominant side. The motor cortex corresponding to the dominant limb may have more extensive corticospinal projections (Tam et al., 2019). Studies show asymmetrical distribution in the cortico-spinal tracts, with the dominant upper limb potentially influencing the density and efficacy of neural pathways (Jaspers et al., 2016).

Functional studies investigated the effects of leg dominance on postural control during challenging balance exercises (Promsri et al., 2020; Kadri et al., 2021). The non-dominant leg exhibited tighter control in diagonal-plane movements, characterized by anterolateral hip strategies, suggesting it requires more precise control in complex movements involving multiple joints and muscle groups (Promsri et al., 2020). Another study found that athletes in asymmetric sports showed more pronounced differences between their dominant and non-dominant legs, with the dominant leg more affected by fatigue and increased postural instability (Kadri et al., 2021). These results align with our findings of an asymmetric result of lateral tES and a pronounced stimulation effect on the side of the non-dominant leg.

The asymmetry in bilateral evoked potentials with tES has been attributed to both anatomical and functional peculiarities of individual muscles or muscle groups (Sayenko et al., 2015). The authors of the study suggested in the discussion that the asymmetry may be related to leg dominance, but they did not record leg dominance in the experiments and recommended this as an additional measure in future experiments.

In both studies with tES applied at L1-L2 and at T11-T12 vertebrae levels, we obtained significant postural response only during the midline and left roots tES. We attribute this effect to differences in anatomical and functional asymmetry associated with leg dominance. Further research is necessary to establish the main factors for lateral asymmetry in targeted neuromodulation of the SC.

## 5 Limitations and future directions

Due to the limitations of using an anechoic chamber we did not record electro-myography. We see potential in simultaneous recording of postural balance and motion capture system for joint coordination analysis.

## 6 Conclusion

The study examined the modulatory effect of tES applied at the T11-T12 vertebrae level on individuals with different cognitive styles. Cognitive style is coupled to exteroceptive or interoceptive afferent stream that dominates during standing control and plays a crucial role in how individuals respond to postural interventions such as tES. In the absence of visual information, FD individuals demonstrate an “en bloc” operation of stiffening the shoulder-hip unit. With limited exteroceptive information, T11-T12 stimulation improved vertical stability in FD participants, likely due to increased hip joint stiffness. Under the same conditions, stimulation was ineffective in FI participants. The result of spinal root tES was asymmetrical. Stimulation improved stability when applied to the side of the non-dominant leg. Further research on muscle activity, joint coordination modes, and postural strategies is required to assess the spinal modulatory effect of tES.

## Data Availability

The raw data supporting the conclusions of this article will be made available by the authors, without undue reservation.
