# Supplementary material for: Transcutaneous spinal cord stimulation modulates quiet standing in healthy adults: stimulation site and cognitive style matter
Source: Front Neurosci. 2024 Sep 11;18:1467182. doi: 10.3389/fnins.2024.1467182 (PMC11422384; doi:10.3389/fnins.2024.1467182)
Supplement: Supplementary file 1 [file Data_Sheet_1.docx]

Supplementary Material

**Table S1.** Analysed center of pressure (CoP) parameters

| Parameter | Definition | Formula |
| --- | --- | --- |
| Length of the CoP trajectory along the frontal axis | Length of the frontal component of the CoP signal | 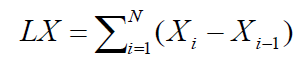 |
| Length of the CoP trajectory along the sagittal axis | Length of the sagittal component of the CoP signal | 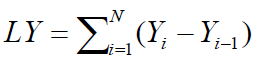 |
| Linear velocity along the frontal axis | The average projection of the linear velocity in the frontal plane | 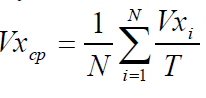 |
| Linear velocity along the sagittal axis | The average projection of the linear velocity in the sagittal plane | 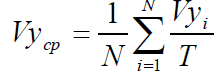 |
| RMSD along the frontal axis | Root mean square deviation of the CoP position along the frontal axis | 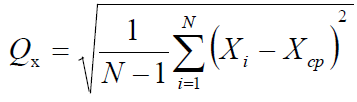 |
| RMSD along the sagittal axis | Root mean square deviation of the CoP position along the sagittal axis | 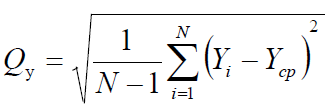 |
| Confidence ellipse area | The main part of the area occupied by the CoP without so-called loops and accidental outliers | 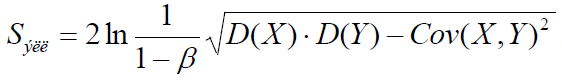 |

X_i_. Y_i_ – CoP coordinates in time

N – number of counts

β –probability that the point of the statokinesiogram hits into the ellipse (β = 0.9).

D(X), D(Y) – corresponding component dispersion

**Table S2.** Length of the CoP trajectory along the frontal axis by participant’s cognitive type and experimental condition (mm).

| **participants** | **control** | **left tES** | **midline tES** | **right tES** |
| --- | --- | --- | --- | --- |
| FD (N=18) | 178 ± 46 | 141 [97; 199] | 128 [100; 183]* | 153 [107; 193] |
| FI (N=18) | 109 [76; 196] | 136 ± 62 | 98 [71; 223] | 130 [64; 203] |
| All (N=36) | 156 ± 53 | 149 ± 45 | 124 [96; 196]^#^ | 151 ± 53 |

*p = 0.02, Z = -2.53; #p = 0.09, Z = -1.65 compared to control condition.

**Table S3.** Length of the CoP trajectory along the sagittal axis by participant’s cognitive type and experimental condition (mm).

| **participants** | **control** | **left tES** | **midline tES** | **right tES** |
| --- | --- | --- | --- | --- |
| FD (N=18) | 263 [160; 313] | 222 [160; 266]* | 240 [132; 271] | 249 [146; 284] |
| FI (N=18) | 161 [144; 249] | 165 [145; 248] | 165 [135; 247] | 164 [127; 256] |
| All (N=36) | 243 [161; 294] | 210 [164; 261]^#^ | 220 [164; 267] | 224 [151; 284] |

*p = 0.02, Z = -2.61; #p = 0.07, Z = -1.75 compared to control condition.

**Table S4**. Ellipse area by participant’s cognitive type and experimental condition (mm^2^)

| **participants** | **control** | **left tES** | **midline tES** | **right tES** |
| --- | --- | --- | --- | --- |
| FD (N=18) | 187 [51; 346] | 136 [42; 201]^*^ | 164 [57; 300] | 143 [43; 353] |
| FI (N=18) | 144 [67; 205] | 131 [76; 193] | 95 [78; 230] | 112 [47; 225] |
| All (N=36) | 172 [76; 248] | 131 [83; 198]^#^ | 152 [82; 247] | 132 [57; 277] |

*p = 0.01, Z = -2.11; #p = 0.06, Z = -1.85 compared to control condition.

**Table S5.** Linear velocity along the frontal axis by participant’s cognitive type and experimental condition (mm/sec).

| **participants** | **control** | **left tES** | **midline tES** | **right tES** |
| --- | --- | --- | --- | --- |
| FD (N=18) | 5.7 ± 1.2 | 4.7 [3.2 ;6.3] | 4.1 [3.3; 5.9]^*^ | 5.0 [3.4; 6.3] |
| FI (N=18) | 3.6 [2.4; 6.3] | 4.1 [2.5; 5.6] | 3.2 [2.2; 7.3] | 4.3 [2.0; 6.6] |
| All (N=36) | 5.0 ± 1.7 | 4.6 [3.3; 6.0] | 4.0 [3.1; 6.4] | 5.0 [3.3; 6.4] |

*p = 0.02, Z = -2.46 compared to control condition.

**Table S6.** Linear velocity along the sagittal axis by participant’s cognitive type and experimental condition (mm/sec).

| **participants** | **control** | **left tES** | **midline tES** | **right tES** |
| --- | --- | --- | --- | --- |
| FD (N=18) | 8.7 [5.1; 10.2] | 7.3 [5.3 ;8.6]^*^ | 7.9 [4.3; 8.7] | 7.9 [4.7; 9.3] |
| FI (N=18) | 5.3 [4.6; 8.2] | 5.3 [4.6; 8.2] | 5.4 [4.4; 8.0] | 5.5 [4.0; 8.2] |
| All (N=36) | 8.0 [5.2; 9.6] | 6.8 [5.2; 8.6]^#^ | 7.3 [5.2; 8.6] | 7.3 [4.9; 9.3] |

*p = 0.02, Z = -2.73; #p = 0.07, Z = -1.78 compared to control condition.

**Table S7.** RMSD along the frontal axis by participant’s cognitive type and experimental condition (mm).

| **participants** | **control** | **left tES** | **midline tES** | **right tES** |
| --- | --- | --- | --- | --- |
| FD (N=18) | 3.8 ± 1.1 | 3.1 ± 1.0^*^ | 3.3 ± 1.4 | 4.0 ± 2.1 |
| FI (N=18) | 2.4 [1.7; 3.5] | 2.7 ± 1.0 | 3.0 ± 1.4 | 2.8 ± 1.4 |
| All (N=36) | 3.4 [1.8; 4.2] | 3.0 ± 1.0^#^ | 2.9 [2.1; 4.0] | 3.0 [1.8; 4.2] |

*p = 0.01, T = -3.29; #p = 0.03, Z = -2.19 compared to control condition.


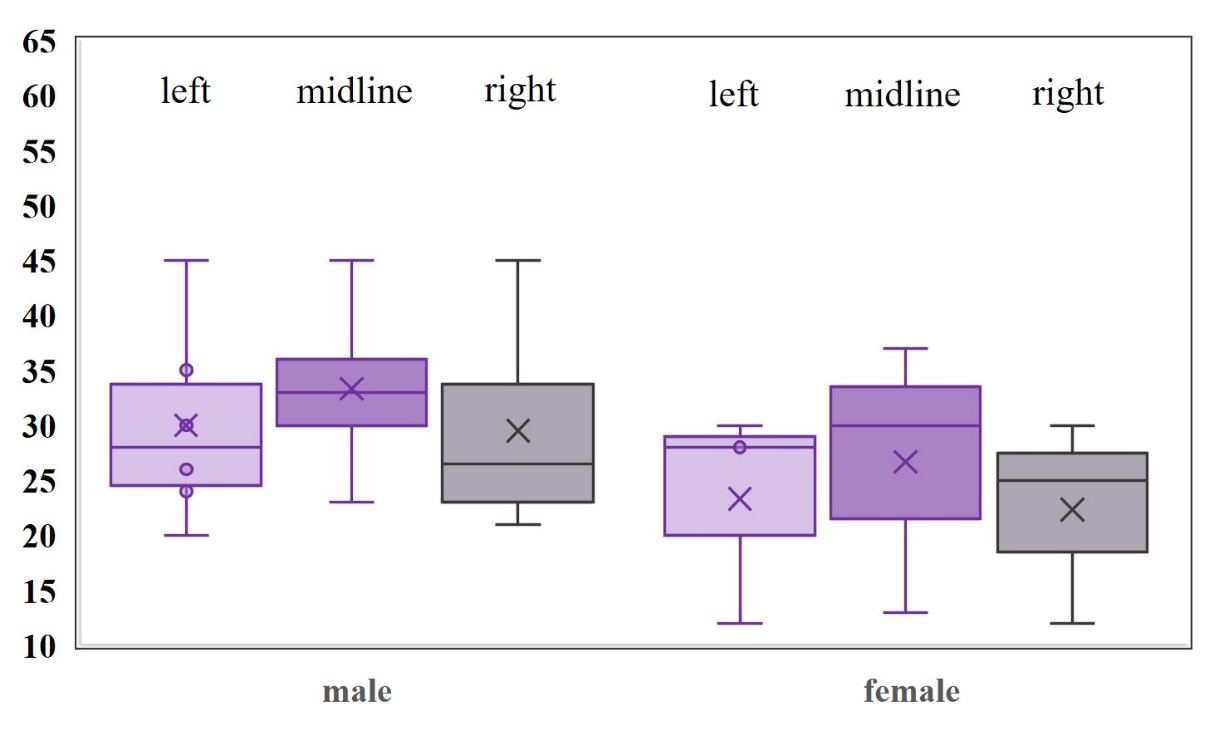


**Figure S1.** Individual variability of current instensity in FD group by sex. Number of participants = 9, 6 males and 3 females.


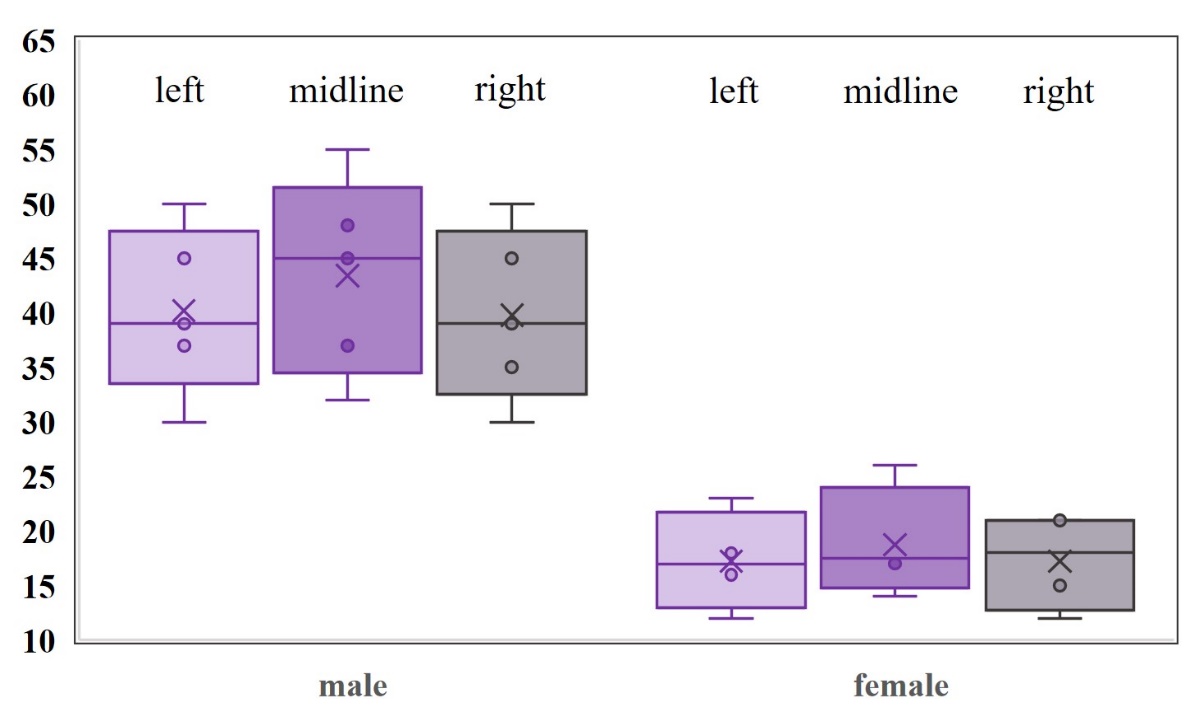


**Figure S2.** Individual variability of current instensity in FI group by sex. Number of participants = 9, 5 males and 4 females.
